# Supplementary figures and images for: Resveratrol inhibits bladder cancer proliferation by targeting the AURKA/STAT3 axis: From computational analysis to experimental validation
Source: PLoS One. 2026 Jan 30;21(1):e0342162. doi: 10.1371/journal.pone.0342162 (PMC12857983; doi:10.1371/journal.pone.0342162)

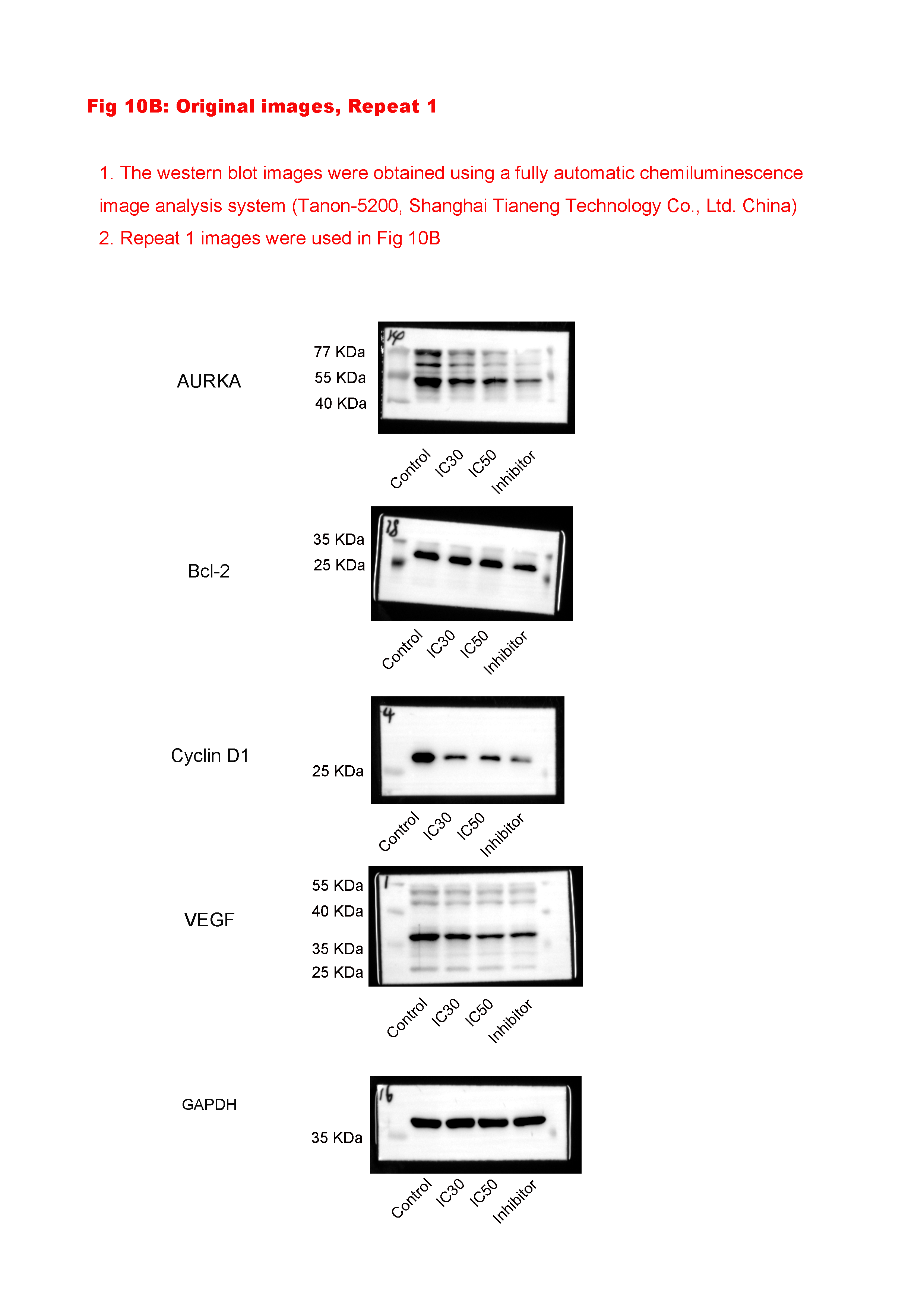

Supplement: S1 Fig — (TIF) [file pone.0342162.s001.tif]

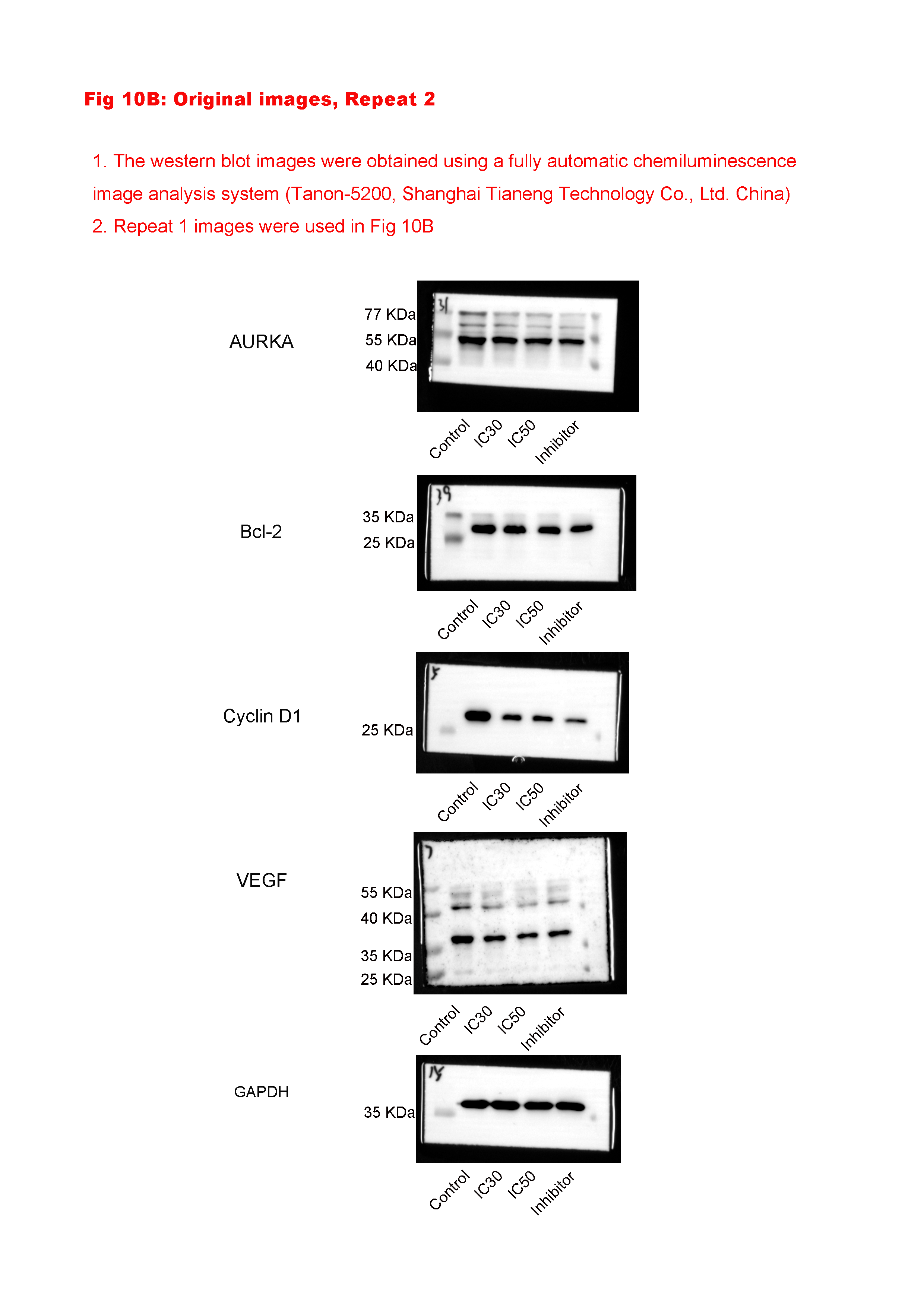

Supplement: S2 Fig — (TIF) [file pone.0342162.s002.tif]

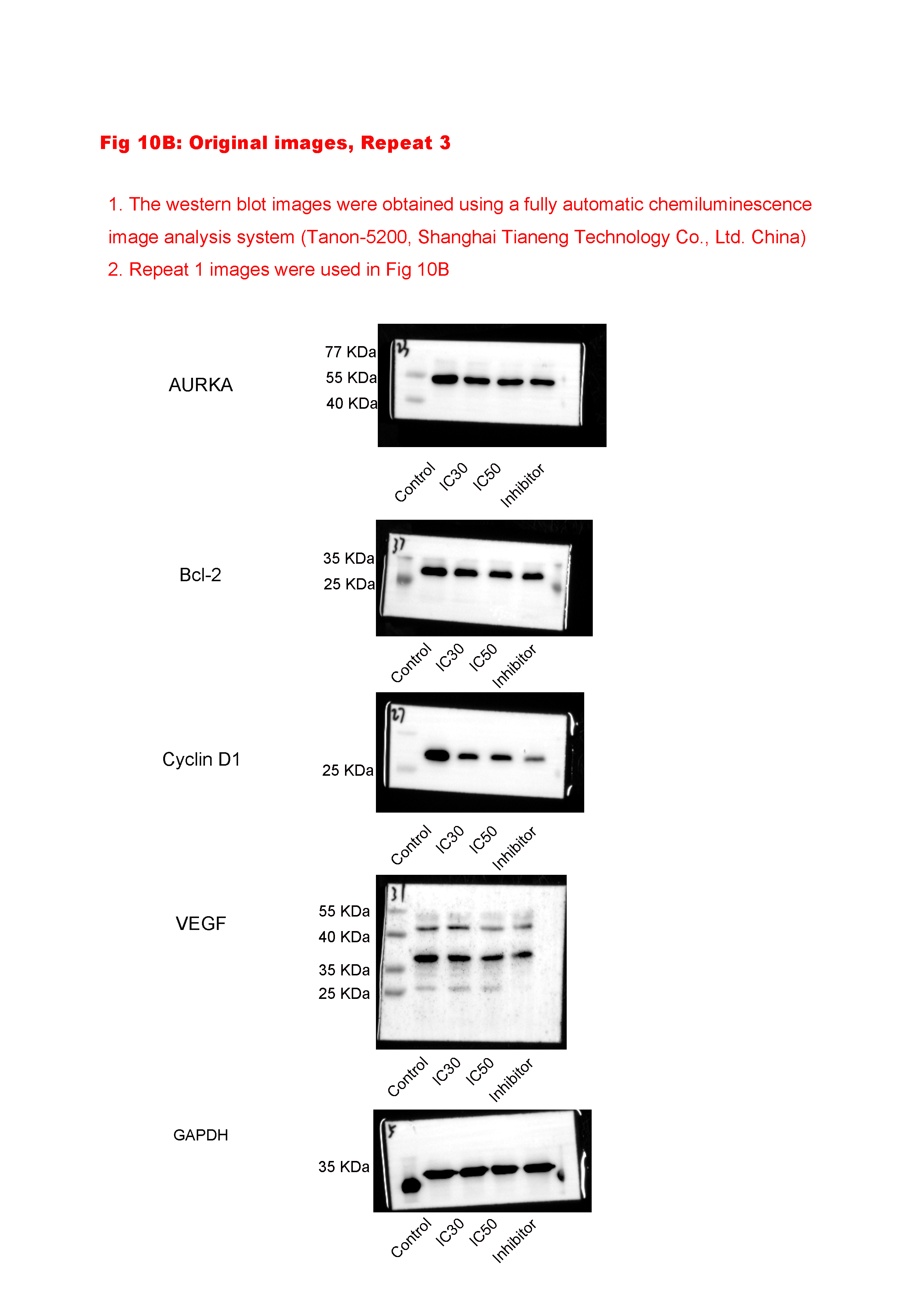

Supplement: S3 Fig — (TIF) [file pone.0342162.s003.tif]

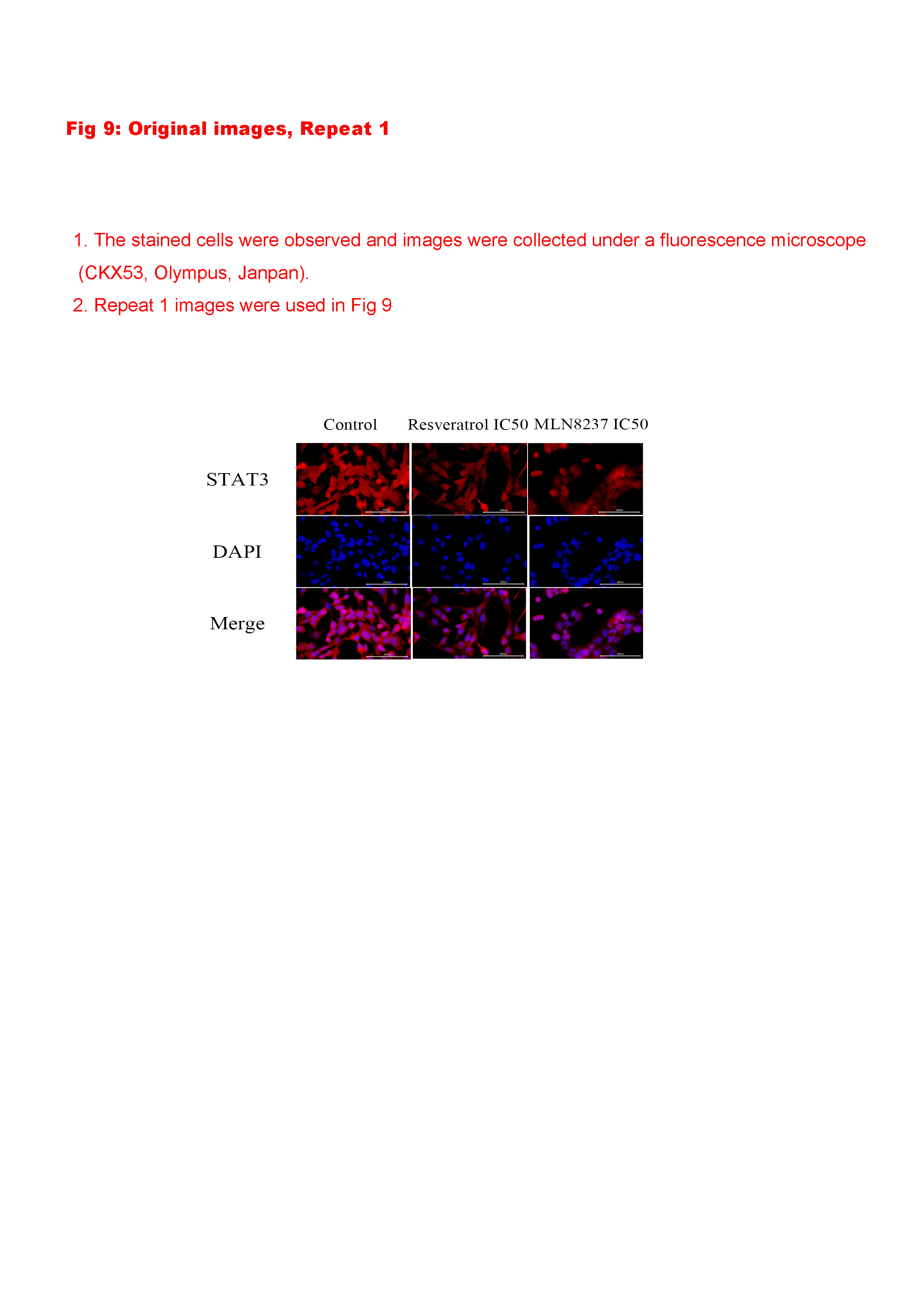

Supplement: S4 Fig — (TIF) [file pone.0342162.s004.tif]

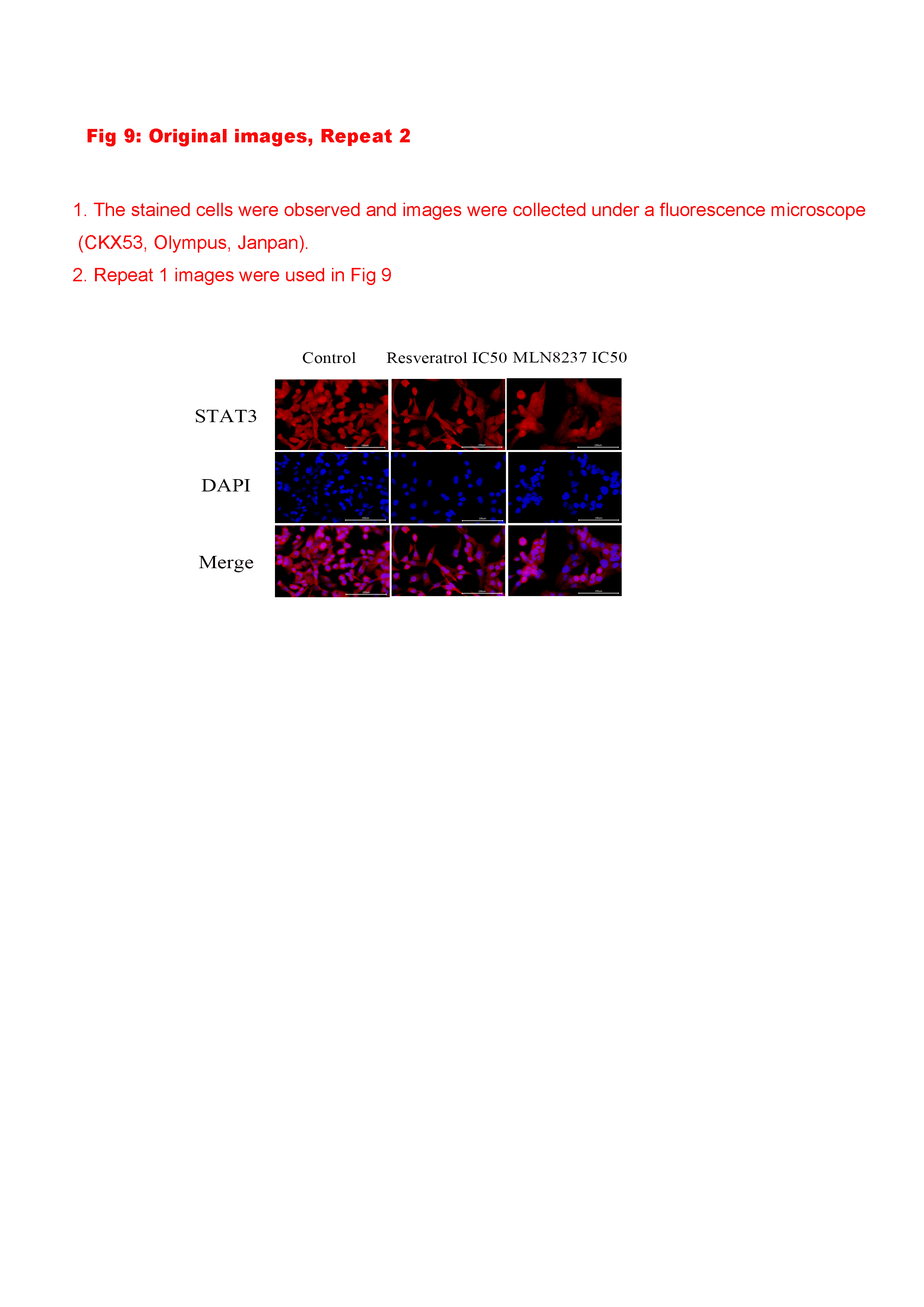

Supplement: S5 Fig — (TIF) [file pone.0342162.s005.tif]

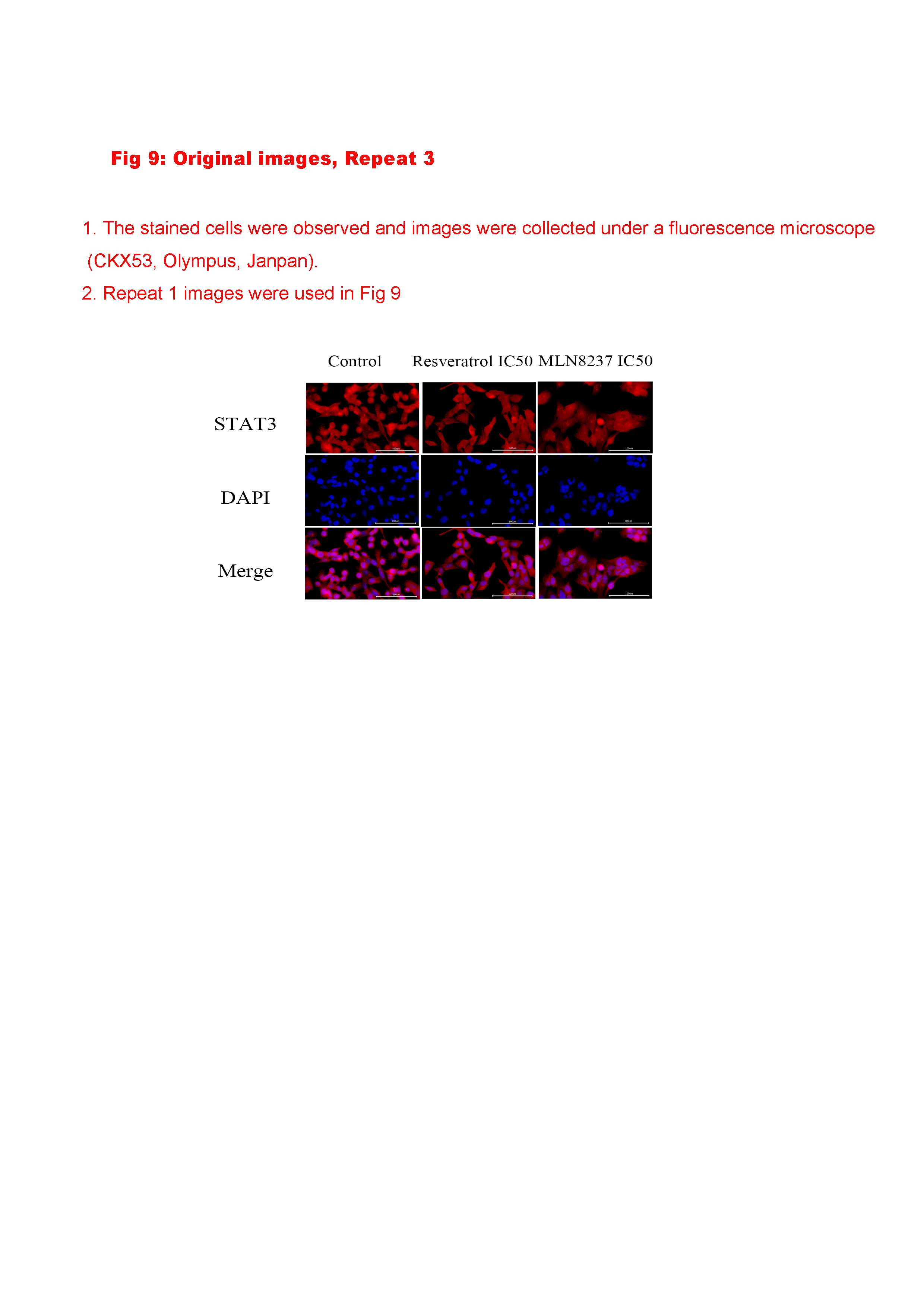

Supplement: S6 Fig — (TIF) [file pone.0342162.s006.tif]

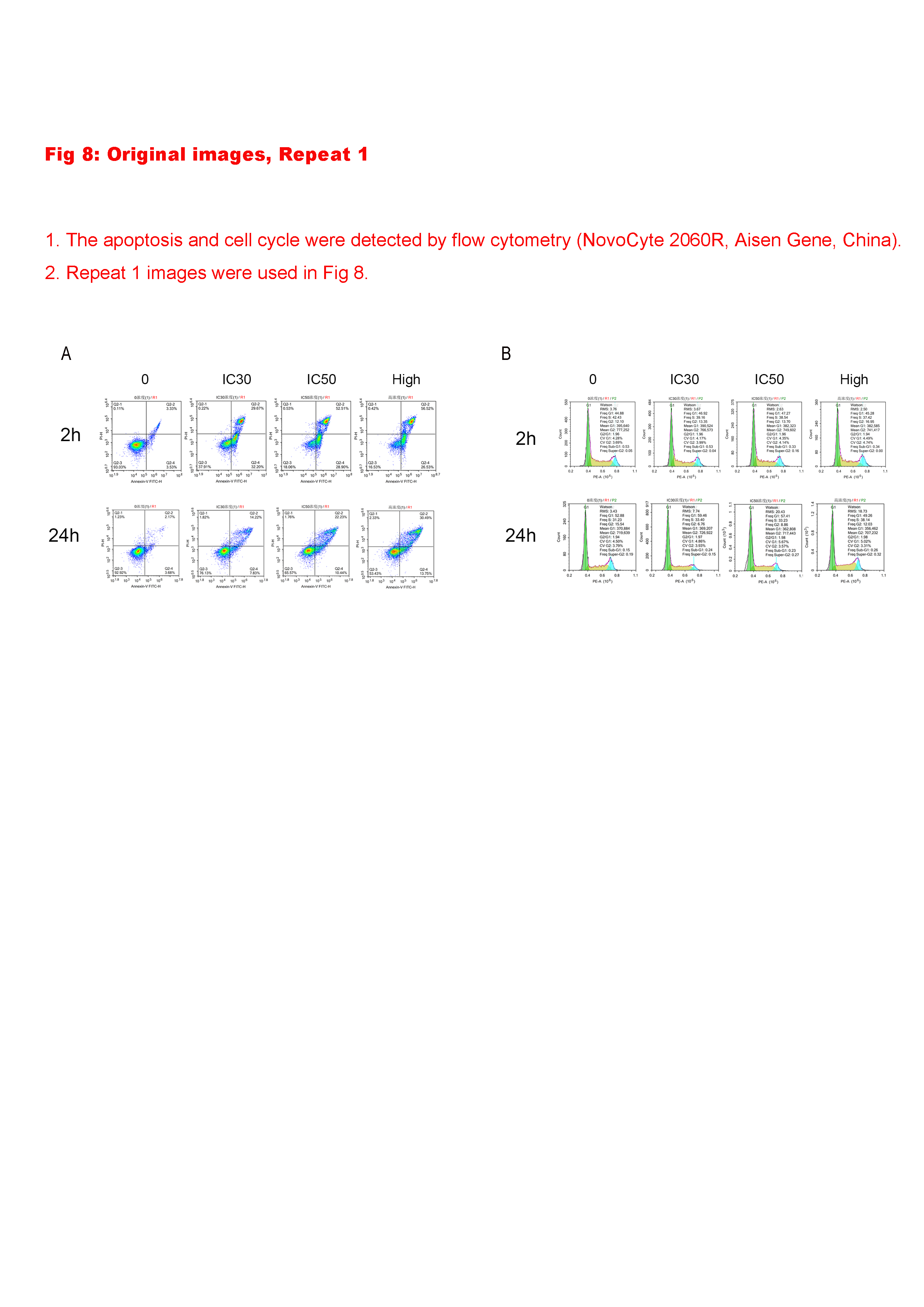

Supplement: S7 Fig — (TIF) [file pone.0342162.s007.tif]

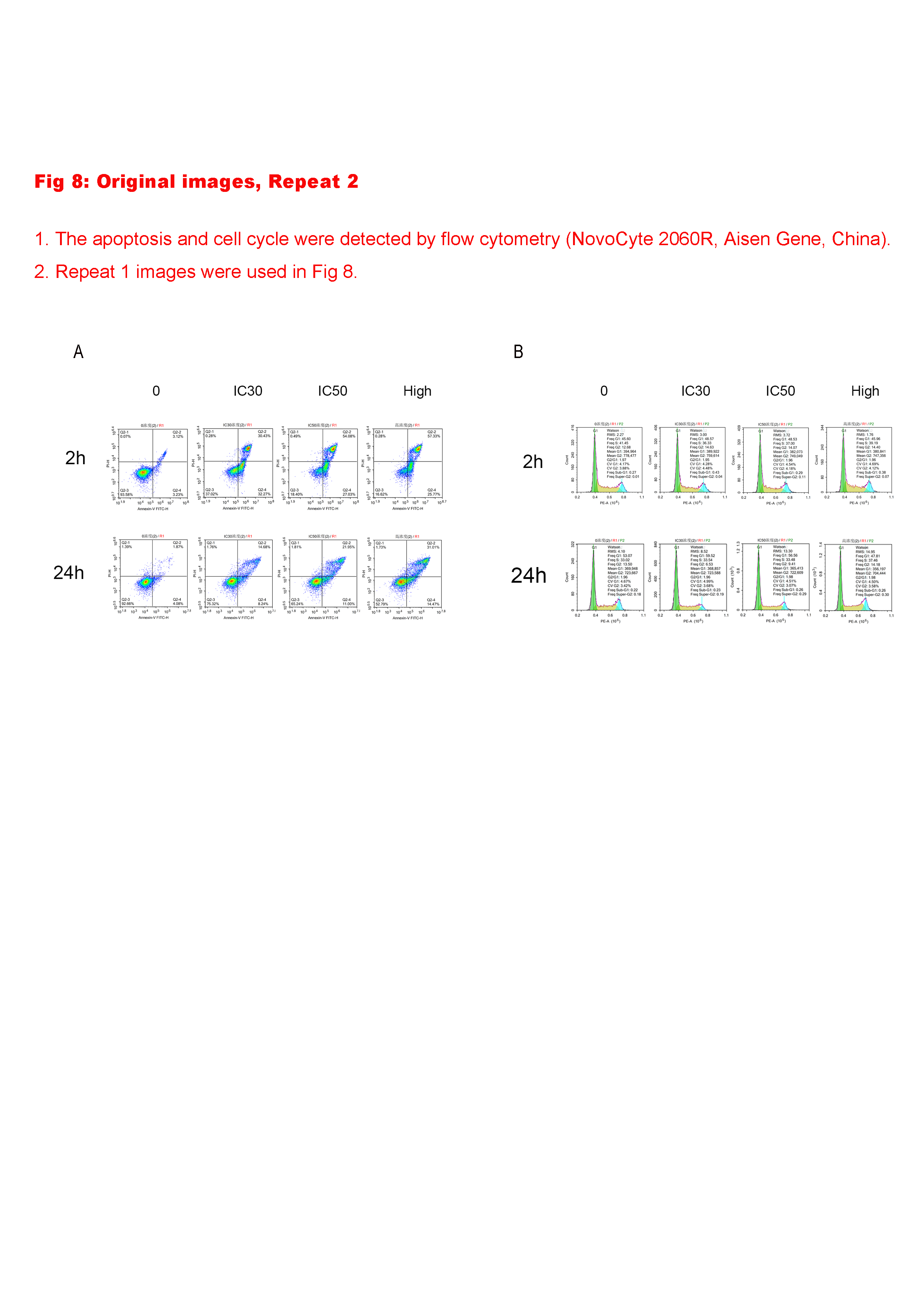

Supplement: S8 Fig — (TIF) [file pone.0342162.s008.tif]

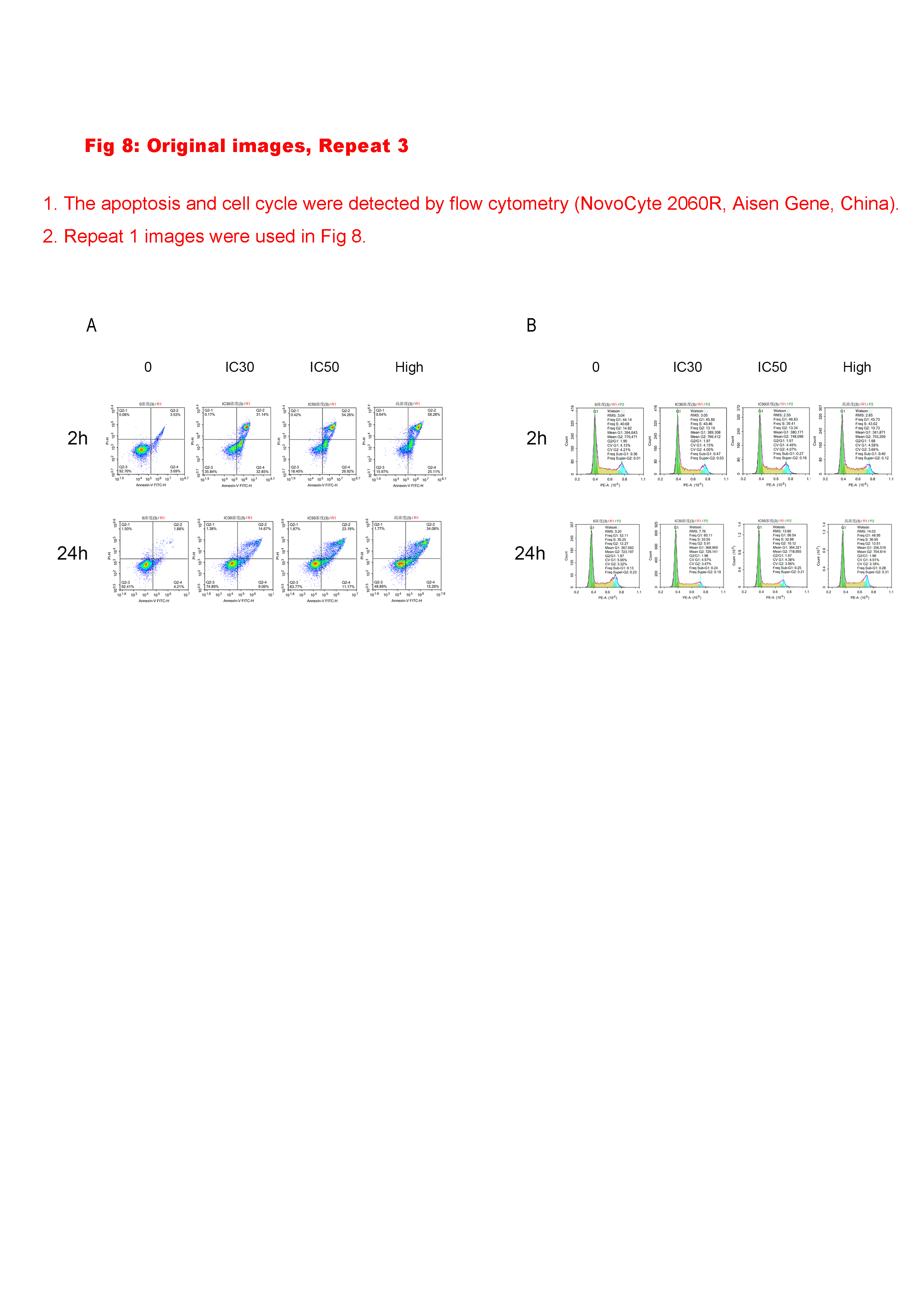

Supplement: S9 Fig — (TIF) [file pone.0342162.s009.tif]
