# Supplementary material for: Resveratrol inhibits bladder cancer proliferation by targeting the AURKA/STAT3 axis: From computational analysis to experimental validation
Source: PLoS One. 2026 Jan 30;21(1):e0342162. doi: 10.1371/journal.pone.0342162 (PMC12857983; doi:10.1371/journal.pone.0342162)
Supplement: Attachment — Submitted filename: Response to Reviewers.docx [file pone.0342162.s018.docx]

Response to Reviewers

Dear Editors and Reviewers,

We sincerely appreciate all the editors and professional reviewers for the valuable feedback which has greatly improved the quality of our manuscript [No.: PONE-D-25-45593]. We carefully reviewed the formatting requirements of PLOS ONE and made thorough revisions. Additionally, we provided the raw data and images in supporting information files and updated the ORCID identifier (0009-0001-5448-2012). Following reviewers’ valuable suggestions, the detailed point-by-point response is as below.

**Reviewer #1:**

**Comment:**

Authors need to provide the original raw data in triplicates for Figures 6 and 7, images should be uncropped and unedited, in original form as there seems to be some kind of manipulation (apparently) in the said figures, please provide the required original data.

**Response：**

Thank you for your kind comments on our manuscript. We have provided the original images and data of Figure 6 and Figure 7 in the supplementary materials as requested. The raw data file was named as " S5 Appendix". We created the original images of the above two pictures into separate PDF files and named " S2_raw_images" and " S3_raw_images". Furthermore, due to the revision of our manuscript, the original pictures 6 and 7 were respectively redefined as Fig 8 and 9.

**Reviewer #2:**

**Comment:**

1. Lines 113-115: The number of normal samples is insufficient. Subsequent analyses based on these 134 samples are not presented; supplementation is recommended.

**Response：**

Thank you very much for your valuable suggestions. We agree that increasing the pool of normal samples will strengthen the research's rigor and comprehensiveness. To address the issue of insufficient normal samples, when using the GEPIA tool based on the TCGA database to analyze the differentially expressed genes (DEGs), we associated the TCGA normal and GTEx data, thereby increasing the sample size of the normal group. Meanwhile, we further utilized the TCGA database to conduct differential gene screening from the perspective of immune infiltration, which addressed the issue of insufficient evidence for DEGs in bioinformatics analysis. We hope that our response will be approved by you. The supplementary content of manuscript is as follows.

“Lines 145-147: The expression level of specific DEGs was analyzed using the MIBC, matched TCGA normal and Genotype-Tissue Expression (GTEx) group data.

Lines 112-116: These data were used to identify immune cell infiltration signatures between MIBC and normal bladder samples. Additionally, the correlation between DEGs and immune infiltration cells (IICs) was examined by the Tumor Immune Estimation Resource (TIMER) database analysis tool.”

**Comment:**

2. Lines 167-169: Ten docking replicates are relatively few; increasing the number is suggested.

**Response：**

We sincerely appreciate the reviewer's insightful suggestion regarding the binding pocket radius. As recommended, we have expanded the docking search space from 10 to 25 using Autodock software. After our analysis, we found that among the 10 and 25 results, the existing conformations we selected remain the optimal ones, with the energy remaining at a relatively low level. Moreover, the reaction path and reaction distance are all in a reasonable state. Considering your valuable comments, we changed the number of dockings to 25 and carried out a more detailed interpretation of the results.Thank you for your kind suggestions for improving our research methods.

**Comment:**

3. Lines 170-198: Inconsistent formatting is present.

**Response：**

Thank you for your suggestions on improving the format of our manuscript. We re-read the submission guidelines of PLOS ONE and set the font to Arial. The font size for the first-level headings is 18 points and the second-level headings is 16 points in bold. The main text it is 12 points with double line spacing.

**Comment:**

4. Lines 260-265: The fold-change of core genes in the GSE130598 transcriptome dataset is not shown; supplementation is recommended.

**Response：**

Based on your review comments, we have added the fold-change information of the core genes in the manuscript. The specific content of manuscript is as follows.

“Lines 336-340: The top ten DEGs, including MTOR, CDK1, FYN, CHEK1, CDK2, CHEK2, ABL1, AURKA, AURKB, and PLK1, with a connective degree of ≥ 24 were identified as hub genes. The fold-changes of these ten core genes were respectively 79.78, 584.75, -308.59, 161.25, 167.15, 90.28, -338.23, 132.12, 444.21, 304.87.”

**Comment:**

5. Lines 266-272 and Figure 3: The number of samples in the normal group is too low, potentially limiting representativeness and the ability to accurately distinguish differences from the tumor group. Furthermore, the differential analysis of core genes in the external dataset does not present fold-change values, and stability or sensitivity assessments, such as ROC analysis, are lacking. The potential markers ABL1, AURKA, CDK1, and FYN, as mentioned by the authors, are not sufficiently substantiated. Was quantitative comparison performed for Figure 3B?

**Response：**

Thanks for your insightful comments which will have substantially strengthened the methodological rigor of our study. In order to address the issues of insufficient normal group, lack of sensitivity assessment, and insufficient evidence for potential markers, we firstly associated the GTEx data in the differential gene analysis to compensate for the insufficient normal sample size in the TCGA database. At the same time, |log2FC| ≥ 1 and adjusted-p < 0.05 were considered statistically significant. We also have named the data of 182 DEGs as “S1 Appendix” and placed it in the supplementary materials. All regulated genes were summarized and plotted on a volcano map (Fig 2A). Additionally, our manuscript added a new section on immune infiltration analysis to verify the reliability of potential markers. Due to the addition of manuscript content, we replaced Figure 3B with Figure 5A. We compared the protein expression of the differentially expressed genes (DEGs) screened using the HPA public database. We agree with the reviewer's suggestion of conducting quantitative analysis, which can provide a more objective and quantitative assessment of the differences between genes or proteins in different tissues. Our manuscript also holds that simple bioinformatic analysis is insufficient to verify the existence and practical value of DEGs. Therefore, our study analyzed the correlation between DEGs and immune cells through the TIMER database. We also objectively verified the mRNA expression level and proteomic differences of the AURKA gene through molecular docking models and multi-angle experiments, which were consistent with the HPA screening results. These results remedied the limitations of exploratory research. The added contents in the manuscript are as follows.

“**Lines 149-168:**

**2.4 Correlation between the immune cell infiltration profile and DEGs**

A total of 22 IIC types were collected and analyzed using the CIBERSORT algorithm. In this study, an adjusted-p< 0.05 was defined as the evaluation criterion required to assess the score results of related immune cell groups. After filtration, the data was used for further processing. The Wilcoxon rank-sum test was used to compare the infiltration levels of different immune cell types between MIBC and normal control samples. Survival analysis of specific immune cell subtypes was performed based on the median of various immune cell proportions. “Survival” packages were used to compare the survival rates of patients with high and low expression of immune cells.

**2.5 Protein expression of the hub genes and tumor-infiltrating immune cell analysis**

Protein expression of the hub genes in MIBC samples was verified using Human Protein Atlas (HPA, https://www.proteinatlas.org) data. The selected results of the hub genes were examined using the Tumor Immune Estimation Resource (TIMER, https://cistrome.shinyapps.io/timer/) database, which was used for systematic analysis of immune infiltrations with six usual types of IICs. The correlation between expression of the screened hub genes and the six IIC types was determined and visualized. The correlation between tumor purity level and each targeted gene was also performed.

**Lines 364-378:**

**3.2 The landscape of immune infiltration in MIBC**

The infiltration of 22 immune cell subpopulations was examined in MIBC tissue, and the difference between tumor and normal tissue was compared using the CIBERSORT algorithm. The proportion of each immune cell type varied significantly between groups (Fig. 4A), and was weakly or strongly correlated with MIBC (Fig. 4B). CD8+ T cells had a stronger positive correlation (Pearson correlation = 0.61) than activated memory CD4+ T cells and a stronger negative correlation (p= -0.56) than resting memory CD4+ T cells. CD8+ T cells also had a moderate negative correlation with M0 macrophages (p= -0.48). These results indicated that the immune response associated with MIBC is a complex cell network that proceeds in a closely regulated manner. Overall survival analysis of the IICs subsets showed that the level of CD8+ T cells (p= 0.027), activated memory CD4+ T cells (p= 0.009), regulatory T cells (Tregs) (p= 0.015) and M0 macrophages (p= 0.005) were significantly associated with patient survival. The Kaplan-Meier curve of significant immune cells is shown in Fig 4C.

Lines 391-397: The correlation between four hub genes and IICs was investigated using the TIMER tool (Fig. 5B). CD8+ T cell infiltration was positively correlated with the expression of ABL1, AURKA, CDK1 and FYN. Meanwhile, AURKA expression correlated positively with dendritic cell infiltration. ABL1 and FYN were significantly correlated with other immune infiltrating cells, including CD4+ T cells, macrophage, neutrophils, and dendritic cells.”

**Comment:**

6. Figure 8 lacks molecular weight markers for proteins. The reason for not detecting STAT3 via Western blot should be explained. Phosphorylated STAT3, a key indicator of pathway activation and functional status, was not assessed; its detection is recommended.

**Response：**

We sincerely thank the reviewer for this crucial and insightful comment. Based on your suggestions, we have refined the molecular weights of the corresponding proteins. The molecular weights of AURKA, Bcl-2, CyclinD1, VEGF and GAPDH are 46KD, 26KD, 34KD, 35KD and 36KD respectively. We agree that the detection of phosphorylated STAT3 (p-STAT3) is a critical piece of evidence for evaluating the functional status of the JAK-STAT pathway. We acknowledge that our original manuscript did not include p-STAT3 data, and we appreciate this opportunity to elaborate on our findings and provide a more comprehensive scientific rationale.

Our primary aim was to investigate the results of resveratrol's impact on the STAT3 signaling pathway. In our experimental design, we chose to employ immunofluorescence (IF) staining as a key method. Our IF results provided compelling and direct evidence that Res significantly alters the expression level of STAT3. This observation is a strong indicator of functional modulation, as the activity of STAT3 is tightly linked to its nuclear translocation upon activation. Furthermore, to confirm the biological consequences of this modulation, we rigorously analyzed the expression of key downstream target genes of STAT3 at both the mRNA and protein levels. The consistent downregulation of these targets across two different molecular levels (transcriptional and translational) provides robust support for the conclusion that Res effectively inhibits the functional output of the STAT3 pathway.

We fully agree that p-STAT3 is the gold-standard biochemical marker for canonical STAT3 activation. Meanwhile, the latest research on the STAT3 pathway has revealed that in addition to the typical tyrosine phosphorylation activation pathway (P-STAT3), there is also a non-phosphorylation pathway (U-STAT3) activated by multiple target genes. Therefore, the process of STAT3 activation is complex and requires continuous follow-up research. We have incorporated this latest discovery into the discussion section of the manuscript. Our IF analysis offers a complementary perspective, presenting the results of the function of the STAT3 pathway in the cellular environment. We are grateful for the reviewer's guidance, which will undoubtedly enhance the depth and completeness of our manuscript.

**Comment:**

7. Inconsistent font styles and non-uniform formatting are observed in figures and tables.

**Response：**

We carefully reviewed the formatting requirements of PLOS ONE and made modifications to the font styles and formats of the figures and tables. We are grateful for the reasonable suggestions provided by the reviewer.

**Comment:**

8. Incorporation of in vivo animal experiments to validate drug effects is recommended.

**Response：**

We sincerely thank the reviewer for this valuable and constructive suggestion. We fully acknowledge that in vivo animal experiments represent the gold standard for preclinical validation and are an essential step towards translating mechanistic findings into potential therapeutic applications. We agree that such experiments would undoubtedly strengthen the overall impact of our study. However, we would like to clarify the primary scope and contribution of our current work, and elaborate on how our existing multi-faceted approach provides a robust and scientifically sound basis for our conclusions. Our study was specifically designed as a mechanistic investigation to explore how Resveratrol might exert its inhibitory effects on bladder cancer cells.

Here, we outline the rationale for our current experimental strategy:

**1.Defining the Scope of Our Study**

The central goal of our manuscript is to propose and elucidate a plausible molecular mechanism for Resveratrol's action in bladder cancer. Our study addresses the question of "how" at a mechanistic level, which is distinct from a therapeutic development study that asks "to what extent does it work in a living organism?" Our findings are intended to provide a solid foundation for future, more applied research.

**2. A Rigorous Multi-Disciplinary Evidence Chain**

We believe that our combination of in vitro cellular assays, bioinformatic analysis, and molecular docking, while not including in vivo models, forms a comprehensive and internally consistent argument for our proposed mechanism. Specifically, we supplemented the relevant analysis of immune infiltration in bioinformatics and the data of differentially expressed genes. Our study inferred DEGs from multiple large databases such as GEO and TCGA, thereby enhancing the reliability of bioinformatics analysis. Subsequently, we confirmed multiple binding sites of Res and AURKA through computational molecular simulation docking model. Then, we conducted a variety of in vitro experiments including cell apoptosis and cycle detection, fluorescence immunoassay, qPCR and WB. Together, these three lines of evidence create a powerful, multi-faceted story that moves from patient data (bioinformatics) to a potential direct target (docking) and finally to a functional cellular outcome (in vitro assays). This integrated approach provides a compelling and self-contained argument for our proposed mechanism.

We view animal experiments as a crucial and exciting next phase of this research line-one that builds upon the mechanistic foundation we have carefully established here. To further express our gratitude for your valid point, we have revised the "Discussion" section of our manuscript. Based on the research hotspots of resveratrol, we have clarified feasible solutions to the problem of low utilization rate of resveratrol. We could construct a stable and efficient nano-delivery system for resveratrol, which will provide broad research prospects for subsequent in vivo experiments and clinical studies. “Lines 604-619: It is undeniable that there are still some deficiencies in our research. The latest research on the STAT3 pathway has revealed that in addition to the typical tyrosine phosphorylation activation pathway (P-STAT3), there is also a non-phosphorylation pathway (U-STAT3) activated by multiple target genes [51]. Although our study indicates that Res inhibits the AURKA/STAT3 axis in bladder cancer cells, the exact molecular mechanism of STAT3 activation mediated by AURKA still needs to be further elucidated. At the same time, our current experimental verification is limited to a single bladder cancer cell line (UMUC-3). Future researches should expand to multiple cell lines representing different molecular subtypes and verify the universality of our findings by constructing overexpressed or low-expressed cells. Moreover, Res currently has many defects such as poor stability, low bioavailability and so on [52]. However, along with the continuous development of various encapsulation methods, it will provide broad research prospects for subsequent in vivo experiments and clinical studies through preparing stable and efficient Res nanodelivery systems [53, 54].” We are grateful to the reviewer for pushing us to think more deeply about the translational potential of our research.

**Comment:**

9. Direct binding was not confirmed through molecular interaction experiments such as co-immunoprecipitation (Co-IP), surface plasmon resonance, or molecular docking; instead, functional assays were relied upon to infer interaction. This weakens the mechanistic rigor; incorporating such experiments is advised.

**Response：**

We are extremely grateful to the reviewer for this insightful and constructive comment，and we appreciate the opportunity to address this important point regarding the mechanistic link between Resveratrol and AURKA. Our reviewer correctly identifies the ideal experimental approaches for confirming direct binding. We wish to provide a detailed explanation of our rationale and present additional supporting evidence.

We agree that Co-IP and Surface Plasmon Resonance (SPR) are the standards for validating protein-protein and protein-ligand interactions, respectively. However, the application of these techniques to our study currently presents insurmountable and technical hurdles in some cases. The technique of Co-IP is specifically designed to detect stable protein-protein complexes. As Res is a small molecule ligand, it does not form a stable stoichiometric complex with AURKA that can be immunoprecipitated as a single entity. While SPR is ideal for small molecule binding, its successful application requires high quantities of ultra-purified, active, and properly folded protein. The expression and purification of full-length, functional AURKA at the purity and in the quantities required for high-quality SPR analysis has proven to be a major bottleneck in many laboratories, including our own. We are actively working to overcome this challenge for future studies.

To further express our gratitude for your opinion, we have strengthened our argument by providing a more in-depth analysis of our molecular docking results. The following text has been added to the Results section:

“Lines 423-435: The results revealed that the molecular docking analysis identified 16 key interacting residues between Res and AURKA, which include critical residues for catalytic activity and allosteric regulation. Notably, the catalytic core residue Asp274 forms both hydrogen bonds and hydrophobic interactions with Resveratrol, acting as a 'dual-lock' to stabilize the conformation of the activation loop. Furthermore, Gly145 forms a backbone hydrogen bond with Res, inducing a steric effect that promotes a contraction of the binding pocket and physically blocks the ATP entry channel. The residue Ala273 contributes to the binding stability by forming a stable hydrophobic stacking with Res. The synergistic effects of these multiple hydrogen bonds and hydrophobic forces construct a stable regulatory conformation, significantly enhancing the affinity of AURKA for Res and playing a crucial role in maintaining the stability of this complex (Fig 6B).”We thank the reviewer again for the valuable feedback, which has helped us to better articulate the strengths and scope of our investigation.

**Comment:**

10. The molecular basis by which AURKA regulates the STAT3 pathway remains unresolved. Is the AURKA inhibitor MLN8237 highly specific for AURKA? More rigorous approaches, such as AURKA knock-down or over-expression, are recommended to substantiate this finding more convincingly.

**Response：**

We are extremely grateful to your profound and insightful feedback, which has prompted us to further analyze and strengthen the mechanistic basis of our study. We fully acknowledge your point that employing AURKA knockdown or overexpression models would indeed represent a more rigorous approach for mechanistic validation. What we want to explain is that our study confirmed the baseline expression levels of AURKA in our key cell lines (UMUC-3 and SV-HUC-1) using qPCR. This step was crucial to establish the experimental model's validity and ensure the feasibility of our subsequent pharmacological studies. While the current study provides robust evidence using complementary pharmacological tools (Resveratrol and MLN8237), we recognize the value of genetic approaches for definitive causal proof. Therefore, in the discussion section of the article, we have outlined the limitations of the study and the future research plans. We are very grateful for your valuable suggestions.

In line with this commitment to enhancing our evidence, we have performed additional experiments to further elucidate the regulatory effects of both Res and MLN8237 on the AURKA/STAT3 axis. As presented in the newly added Figure 10A, our qPCR results clearly demonstrate that Res significantly suppresses AURKA mRNA expression compared to the control group. This finding provides complementary molecular insight into Resveratrol's mechanism of action, reinforcing our multi-faceted validation strategy. Interestingly, the result showed that MLN8237 can increase the mRNA expression level of AURKA, which may be related to the cell's feedback regulation to compensate for the transcription of AURKA. This result revealed that if the effects of MLN8237 were due to an off-target artifact, we would not expect such a specific and compensatory response at the mRNA level. The fact that the cellular machinery mounts a feedback response to AURKA inhibition (upregulating mRNA) but is ultimately overridden by the potent pharmacological blockade (degrading/inactivating protein) is a hallmark of a specific on-target drug. MLN8237, as an ATP competitive kinase inhibitor, acts on the already synthesized AURKA protein itself. We have also confirmed this through relevant literature and have further elaborated on it in the discussion section. The convergent phenotype—suppression of the STAT3 pathway—can only be logically explained by the common denominator: inhibition of AURKA protein function. Resveratrol achieves this by reducing the amount of AURKA protein available. MLN8237 achieves this by disabling the existing AURKA protein. Both pathways lead to the same functional outcome, proving that AURKA activity is the critical node controlling AURKA/STAT3 axis. Thank you again for your insightful guidance and we will continue to optimize and expand our research based on this valuable input. The revision of the discussion section is as follows:

“Lines 567-603: Studies have shown that the continuous activation of STAT3 can promote the transformation from bladder carcinoma in situ to invasive BCa [42]. The STAT3 signaling pathway can also be involved in the formation of tumor inflammatory microenvironment, which is closely related to tumor progression and malignant transformation [48]. At the same time, Jiao Wu et al. found that Res can control the progression of thyroid cancer by regulating the expression of STAT3 [40]. Additionally, the AURKA inhibitor MLN8237 has been confirmed to inhibit the growth of breast cancer cells by altering the tumor microenvironment and suppressing the AURKA kinase. This is unique as the only agent currently in Phase III clinical trials [49]. Based on above theoretical foundation, the GEO and TCGA large databases were used to analyze and screen out the differentially expressed gene AURKA in bladder cancer from multiple perspectives. Experimental validation via qPCR further demonstrated that AURKA mRNA expression was markedly higher in the UMUC-3 cell line than in the normal SV-HUC-1 cell line. These results underscore the significance of AURKA and justify its selection for further in-depth analysis.

Subsequently, the IC50 concentrations of Res and the AURKA inhibitor MLN8237 were determined. Molecular docking simulations confirmed that Res binds to AURKA at multiple sites, establishing a fundamental basis for the subsequent experiments. Meanwhile, our results demonstrated that Res significantly suppressed the mRNA expression of AURKA, thereby influencing the cell cycle and inducing apoptosis. Furthermore, both Res and MLN8237 were found to effectively inhibit the STAT3 signaling pathway, as evidenced by a significant reduction in STAT3 fluorescence intensity and the downregulation of its downstream target proteins, including VEGF, Bcl-2, and Cyclin D1. Previous studies have shown that AURKA, as a factor regulating the cell cycle and apoptosis, has been found to participate in the drug resistance of breast cancer together with the STAT3 pathway [50]. Building upon this, our study is to link this regulatory network to the clinical challenge of targeted therapy resistance in bladder cancer, thereby proposing a novel scientific hypothesis: AURKA and the STAT3 pathway may act synergistically to mediate this resistance. Consequently, this work not only provides a crucial theoretical foundation and a new direction for future validation of this hypothesis but also offers strong data to support the potential clinical therapeutic strategies, such as monotherapy with Res or combination therapies targeting the AURKA/STAT3 axis, to improve treatment outcomes for bladder cancer patients.”

**Reviewer #3:**

**Comment:**

Title：

1.It is suggested that the title add a description of the core mechanism.

2.The content related to molecular docking in the article is relatively brief. If this aspect is intended to be included in the title, it is recommended to further supplement and deepen the discussion on the molecular docking section.

**Response：**

Thank you for your reasonable suggestions regarding the revision of our title. Based on the research content of our manuscript, we revised the title to " Resveratrol inhibits bladder cancer proliferation by targeting the AURKA/STAT3 axis: From computational analysis to experimental validation". At the same time, we provided a more comprehensive interpretation of the molecular docking results, making the article content more complete. The revised content is as follows:

“Lines 420-435: The lower the free energy of substrate binding, the better the effect of binding. To determine the binding ability of Res with AURKA, this study used discovery studio to analyze the interaction forces of the Res and AURKA complex model. The results revealed that the molecular docking analysis identified 16 key interacting residues between Res and AURKA, which include critical residues for catalytic activity and allosteric regulation. Notably, the catalytic core residue Asp274 forms both hydrogen bonds and hydrophobic interactions with Resveratrol, acting as a 'dual-lock' to stabilize the conformation of the activation loop. Furthermore, Gly145 forms a backbone hydrogen bond with Res, inducing a steric effect that promotes a contraction of the binding pocket and physically blocks the ATP entry channel. The residue Ala273 contributes to the binding stability by forming a stable hydrophobic stacking with Res. The synergistic effects of these multiple hydrogen bonds and hydrophobic forces construct a stable regulatory conformation, significantly enhancing the affinity of AURKA for Res and playing a crucial role in maintaining the stability of this complex (Fig 6B).”

**Comment:**

Abstract：

3.The research objectives in the introduction do not align well with the conclusions, and it is recommended to revise the expression of the research objectives.

**Response：**

We are extremely grateful to your reasonable suggestions regarding our manuscript. In order to ensure that the research objectives in the introduction of abstract keep aligning well with the conclusions. we made the following revisions to the introduction part of the abstract:

“Lines 25-33: Given the high recurrence rate of bladder cancer (BCa) and the significant adverse effects associated with conventional treatments, it is urgent to search for new clinical therapeutic targets and safer natural-derived compounds. Resveratrol (Res) has been demonstrated to exhibit cytotoxicity against various tumors. However, the signaling pathways and targets involved in inhibition of BCa cells still need further exploration. This study aims to investigate the mechanism of Res in Bca via suppression of the AURKA/STAT3 axis, providing important theoretical basis for subsequent further researches on Res for treating BCa.”

**Comment:**

Introduction：

4.“Res shows significant application potential in the prevention and treatment of BCa due to its wide availability, low cost, and clear tumor suppression effect.”It lacks corresponding reference support, and it is recommended to supplement relevant references for verification.

**Response：**

Thank you for your valuable suggestions in improving the content of our manuscript. Based on the article's content, we have added relevant reference and inserted it at the appropriate position.

“Lines 78-80: Res shows significant application potential in the prevention and treatment of BCa due to its wide availability, low cost, and clear tumor suppression effect [14].”

**Comment:**

5.It is suggested to add the current progress of natural medicines in BCA treatment in the preface or discussion section, and appropriately include an explanation of the application of resveratrol in tumors.

**Response：**

Thank you very much for your suggestions regarding the supplementation of natural medicines and the application of resveratrol in tumors. Based on the description of resveratrol in the introduction section of our manuscript, we supplemented the discussion part about the application of natural medicines in bladder cancer and the current status of resveratrol's application in tumors. The detailed content is as follows:

“Lines 525-545: At present, the treatment of MIBC remains a formidable clinical challenge, with high invasiveness leading to a persistently high recurrence and metastasis rate [23]. Relevant studies have shown that the 5-year survival rate of metastatic patients is lower than 15%, which urges us to urgently develop novel and cost-effective therapeutic strategies [24, 25]. Natural compounds, such as curcumin, lycopene, resveratrol, and quercetin, have been explored for their potential in treating various tumors, offering new research directions in oncology [26-29]. Aronia melanocarpa, orinda citrifolia juices, resveratrol and other natural drugs have demonstrated certain anti-tumor effects in bladder cancer [25, 30]. However, it should be noted that although resveratrol first demonstrated anti-metastatic potential as early as 1997, the related research progress remains limited so far. This underscores the critical need to identify its key direct molecular targets and the associated downstream signaling pathways in its anti-tumor effects [31, 32]. Meanwhile, AURKA is considered to have valid biological function and prognostic significance in neuroblastoma, medullary thyroid carcinoma, hepatocellular carcinoma and other tumors [33-36]. As an important serine/threonine kinase, AURKA plays a critical role in the G2/M transition, promoting mitosis and other functions [37]. Although several previous studies have shown that AURKA may be a target gene of BCa, there are relatively few studies on how it affects the progression of BCa cells between natural anti-tumor drugs and related pathways [20, 38].”

**Comment:**

Materials and Methods：

6.It is recommended to supplement the definition to HUB genes in Section 2.2 of the materials.

**Response：**

In accordance with the requirements, we have supplemented the definition of HUB genes. “Lines 139-141: Hub genes refer to nodes representing proteins that exhibit the high connectivity and they play critical roles in coordinating biological processes and serve as key regulators within the network.” Thank you for your reasonable suggestions in improving our manuscript.

**Comment:**

Result：

7.Western blot experiment suggested to construct the overexpression plasmid of AURKA, transfect it into SV-HUC-1 or bladder cancer cell line with low expression of AURKA, and then give Res to observe whether Res can reverse the increase of p-STAT3 caused by overexpression of AURKA and accelerate cell proliferation, so as to verify whether AURKA is the direct target of Res.

**Response：**

We are extremely grateful to your profound and insightful feedback, which has prompted us to further analyze and strengthen the mechanistic basis of our study. We fully acknowledge your point that employing AURKA overexpression models would indeed represent a more rigorous approach for mechanistic validation. What we want to explain is that our study confirmed the baseline expression levels of AURKA in our key cell lines (UMUC-3 and SV-HUC-1) using qPCR. This step was crucial to establish the experimental model's validity and ensure the feasibility of our subsequent pharmacological studies. While the current study provides robust evidence using complementary pharmacological tools (Resveratrol and MLN8237), we recognize the value of genetic approaches for definitive causal proof. Therefore, in the discussion section of the article, we have outlined the limitations of the study and the future research plans. We are very grateful for your valuable suggestions.

In our experimental design, we chose to employ immunofluorescence (IF) staining as a key method. Our IF results provided compelling and direct evidence that Res significantly alters the expression level of STAT3. We fully agree that p-STAT3 is the gold-standard biochemical marker for canonical STAT3 activation. Meanwhile, the latest research on the STAT3 pathway has revealed that in addition to the typical tyrosine phosphorylation activation pathway (P-STAT3), there is also a non-phosphorylation pathway (U-STAT3) activated by multiple target genes. Therefore, the process of STAT3 activation is complex and requires continuous follow-up research. We have incorporated this latest discovery into the discussion section of the manuscript. Our IF analysis offers a complementary perspective, presenting the results of the function of the STAT3 pathway in the cellular environment.

In line with this commitment to enhancing our evidence, we have performed additional experiments to further elucidate the regulatory effects of both Res and MLN8237 on the AURKA/STAT3 axis. As presented in the newly added Figure 10A, our qPCR results clearly demonstrate that Res significantly suppresses AURKA mRNA expression compared to the control group. This finding provides complementary molecular insight into Resveratrol's mechanism of action, reinforcing our multi-faceted validation strategy. Interestingly, the result showed that MLN8237 can increase the mRNA expression level of AURKA, which may be related to the cell's feedback regulation to compensate for the transcription of AURKA. This result revealed that if the effects of MLN8237 were due to an off-target artifact, we would not expect such a specific and compensatory response at the mRNA level. The fact that the cellular machinery mounts a feedback response to AURKA inhibition (upregulating mRNA) but is ultimately overridden by the potent pharmacological blockade (degrading/inactivating protein) is a hallmark of a specific on-target drug. MLN8237, as an ATP competitive kinase inhibitor, acts on the already synthesized AURKA protein itself. We have also confirmed this through relevant literature and have further elaborated on it in the discussion section. “The AURKA inhibitor MLN8237 has been confirmed to inhibit the growth of breast cancer cells by altering the tumor microenvironment and suppressing the AURKA kinase. This is unique as the only agent currently in Phase III clinical trials.” The convergent phenotype—suppression of the STAT3 pathway—can only be logically explained by the common denominator: inhibition of AURKA protein function. Resveratrol achieves this by reducing the amount of AURKA protein available. MLN8237 achieves this by disabling the existing AURKA protein. Both pathways lead to the same functional outcome, proving that AURKA activity is the critical node controlling AURKA/STAT3 axis. Thank you again for your insightful guidance and we will continue to optimize and expand our research based on this valuable input.

**Comment:**

8.Supplement the experiment with "fixed concentration and time as the sole variable" in Section 3.3.

**Response：**

We are extremely grateful to your valuable comments regarding "fixed concentration and time as the sole variable". We fully understand your intention to establish the concentration or time as independent driving factors more strictly. We re-examined the experimental design and data. We think that although our experimental design aimed to evaluate the mechanism by which resveratrol inhibits bladder cancer cells, the existing data proved that under fixed time conditions, concentration is an independent and crucial driving factor for inducing apoptosis, and time (24h vs 2h) is a necessary condition for inducing cycle arrest. Additionally, we reviewed relevant literature and added it to the manuscript, and found that the anti-proliferative effect of resveratrol on bladder cancer cells is indeed concentration-time dependent. Our data are as follows:

Under fixed-time condition, we observed that the apoptosis rates in the IC30 group, IC50 group, and high concentration group were significantly higher than those in the control group, and the apoptosis rate significantly increased with the increase in concentration, presenting a clear dose-dependent relationship. This directly indicates that within the fixed time frame, the ability of resveratrol to induce apoptosis strongly depends on its concentration. The increase in concentration is an independent variable driving the increase in apoptosis rate. Moreover, under 2-hour condition, regardless of the concentration, there was no significant difference in the cell cycle distribution. This strongly proves that within the shorter 2-hour window period, even at high concentrations, resveratrol has not yet induced significant cell cycle arrest. This further emphasizes that time is another key variable for resveratrol to exert its specific biological effects (especially cell cycle arrest). In summary, our current data show that cell apoptosis has a clear concentration dependence in fixed time (Figure 8A). Under fixed time (24h), there is a concentration-dependent change in the cell cycle arrest pattern (Figure 8B). At the same time, time is a necessary condition for inducing cycle arrest. Therefore, we have successfully achieved the research goal under rigorous experimental design (examining concentration gradients at a unified time point and comparing different time points), and clearly revealed the key roles of concentration and time and their synergy. We are very grateful to your valuable comments, which enabled us to conduct detailed thinking and explanation of the research data and help us improve the content of our manuscript.

**Comment:**

9.It is recommended to add a volcano plot for the differential gene results in Section 3.1.

**Response：**

Thank you for your reasonable suggestions regarding the content of our manuscript. As requested, we have added a volcano plot (Fig 2A) to present the results of the differentially expressed genes.

**Comment:**

Discussion：

10.It is recommended to rewrite the discussion section, focusing on the content of the research in this article.

**Response：**

Thank you for your valuable suggestions on improving the quality of our manuscript. We have made significant revisions to the discussion section. In light of the previous revision requirements, we have focused the discussion on elaborating on the research content. The detailed discussion is as follows:

“Lines 524-619:

**4. DISSCUTION**

At present, the treatment of MIBC remains a formidable clinical challenge, with high invasiveness leading to a persistently high recurrence and metastasis rate [23]. Relevant studies have shown that the 5-year survival rate of metastatic patients is lower than 15%, which urges us to urgently develop novel and cost-effective therapeutic strategies [24, 25]. Natural compounds, such as curcumin, lycopene, resveratrol, and quercetin, have been explored for their potential in treating various tumors, offering new research directions in oncology [26-29]. Aronia melanocarpa, orinda citrifolia juices, resveratrol and other natural drugs have demonstrated certain anti-tumor effects in bladder cancer [25, 30]. However, it should be noted that although resveratrol first demonstrated anti-metastatic potential as early as 1997, the related research progress remains limited so far. This underscores the critical need to identify its key direct molecular targets and the associated downstream signaling pathways in its anti-tumor effects [31, 32]. Meanwhile, AURKA is considered to have valid biological function and prognostic significance in neuroblastoma, medullary thyroid carcinoma, hepatocellular carcinoma and other tumors [33-36]. As an important serine/threonine kinase, AURKA plays a critical role in the G2/M transition, promoting mitosis and other functions [37]. Although several previous studies have shown that AURKA may be a target gene of BCa, there are relatively few studies on how it affects the progression of BCa cells between natural anti-tumor drugs and related pathways [20, 38]. In this study, a new therapeutic axis in BCa were elucidated through combing bioinformatics analysis with multi-level experimental verification: Our research not only calculated the amino acid sites where Res combined with AURKA, but also indicated that resveratrol could suppress the proliferation of BCa cells by down-regulating the expression of AURKA. We also discovered that resveratrol may exert its powerful anti-tumor effect by inhibiting the AURKA/STAT3 signaling pathway, thereby inhibiting the proliferation and cell cycle progression of bladder cancer cells. These findings not only provide mechanistic insights into the anti-tumor properties of resveratrol, but also highlight the importance of the AURKA/STAT3 axis as a promising therapeutic target for BCa.

As an important tumor suppressor pathway, the STAT3 pathway has been widely confirmed that it plays a vital role in various common tumors including breast cancer, thyroid cancer, and cervical cancer through inhibiting cancer cell proliferation, metastasis, associating chemotherapy resistance and other aspects [39-41]. VEGF, Bcl-2 and Cyclin D1 have been confirmed to be the target genes which participate in the regulation of the STAT3 pathway [42, 43]. VEGF is involved in the formation of tumor blood vessels in cancer tissues and is an important driver and target in the tumor vascular system [44]. Bcl-2 plays a key role in regulating the homeostasis between autophagy and apoptosis of cells. The overexpression of Bcl-2 is regarded as an independent adverse prognostic factor [45, 46]. Cyclin D1 is a key regulatory factor for the transition from the G1 phase to the S phase in the cell cycle process [47]. Studies have shown that the continuous activation of STAT3 can promote the transformation from bladder carcinoma in situ to invasive BCa [42]. The STAT3 signaling pathway can also be involved in the formation of tumor inflammatory microenvironment, which is closely related to tumor progression and malignant transformation [48]. At the same time, Jiao Wu et al. found that Res can control the progression of thyroid cancer by regulating the expression of STAT3 [40]. Additionally, the AURKA inhibitor MLN8237 has been confirmed to inhibit the growth of breast cancer cells by altering the tumor microenvironment and suppressing the AURKA kinase. This is unique as the only agent currently in Phase III clinical trials [49]. Based on above theoretical foundation, the GEO and TCGA large databases were used to analyze and screen out the differentially expressed gene AURKA in bladder cancer from multiple perspectives. Experimental validation via qPCR further demonstrated that AURKA mRNA expression was markedly higher in the UMUC-3 cell line than in the normal SV-HUC-1 cell line. These results underscore the significance of AURKA and justify its selection for further in-depth analysis.

Subsequently, the IC50 concentrations of Res and the AURKA inhibitor MLN8237 were determined. Molecular docking simulations confirmed that Res binds to AURKA at multiple sites, establishing a fundamental basis for the subsequent experiments. Meanwhile, our results demonstrated that Res significantly suppressed the mRNA expression of AURKA, thereby influencing the cell cycle and inducing apoptosis. Furthermore, both Res and MLN8237 were found to effectively inhibit the STAT3 signaling pathway, as evidenced by a significant reduction in STAT3 fluorescence intensity and the downregulation of its downstream target proteins, including VEGF, Bcl-2, and Cyclin D1. Previous studies have shown that AURKA, as a factor regulating the cell cycle and apoptosis, has been found to participate in the drug resistance of breast cancer together with the STAT3 pathway [50]. Building upon this, our study is to link this regulatory network to the clinical challenge of targeted therapy resistance in bladder cancer, thereby proposing a novel scientific hypothesis: AURKA and the STAT3 pathway may act synergistically to mediate this resistance. Consequently, this work not only provides a crucial theoretical foundation and a new direction for future validation of this hypothesis but also offers strong data to support the potential clinical therapeutic strategies, such as monotherapy with Res or combination therapies targeting the AURKA/STAT3 axis, to improve treatment outcomes for bladder cancer patients.

It is undeniable that there are still some deficiencies in our research. The latest research on the STAT3 pathway has revealed that in addition to the typical tyrosine phosphorylation activation pathway (P-STAT3), there is also a non-phosphorylation pathway (U-STAT3) activated by multiple target genes [51]. Although our study indicates that Res inhibits the AURKA/STAT3 axis in bladder cancer cells, the exact molecular mechanism of STAT3 activation mediated by AURKA still needs to be further elucidated. At the same time, our current experimental verification is limited to a single bladder cancer cell line (UMUC-3). Future researches should expand to multiple cell lines representing different molecular subtypes and verify the universality of our findings by constructing overexpressed or low-expressed cells. Moreover, Res currently has many defects such as poor stability, low bioavailability and so on [52]. However, along with the continuous development of various encapsulation methods, it will provide broad research prospects for subsequent in vivo experiments and clinical studies through preparing stable and efficient Res nanodelivery systems [53, 54].”

Thank you for your guidance and support. Wishing you continued success in your work and life. We look forward to your further feedback.
