# Supplementary material for: Resveratrol inhibits bladder cancer proliferation by targeting the AURKA/STAT3 axis: From computational analysis to experimental validation
Source: PLoS One. 2026 Jan 30;21(1):e0342162. doi: 10.1371/journal.pone.0342162 (PMC12857983; doi:10.1371/journal.pone.0342162)
Supplement: Attachment — Submitted filename: Response to Reviewers.docx [file pone.0342162.s019.docx]

**Point-to-point** **responses to comments**

**Comment:**

Please note that as per our publication criteria, PLOS ONE requires that all experiments, statistics and other analyses are performed to a high technical standard, described in sufficient detail and adhere to appropriate reporting guidelines and community standards. Conclusions must be presented in an appropriate fashion and be supported by the data (Please see <http://journals.plos.org/plosone/s/criteria-for-publication>).

**Response：**

After receiving the comment, we carefully read the publication criteria of PLOS ONE and have made extensive modifications to our manuscript. We provided a detailed description of the experimental methods and reagents to enable other researchers to reproduce the experiments described. At the same time, all experiments were repeated three times to ensure accuracy. We simultaneously checked the conclusions against the experimental data to ensure that the experimental data could support the conclusions.

**Comment:**

You have not provided us with sufficient detail on the study design and analyses to meet these requirements; in particular, we feel that the Methods section lacks important information, such as details of the Western Blot, flow cytometry and immunofluorescence procedures, as well as detailed information on molecular docking and molecular dynamic simulation experiments.

**Response：**

We really appreciate for the reviewers’ and the Academic Editor’s comments. We have made detailed revisions to the relevant experiments mentioned in the above comment and hope that the correction will meet with approval. The detailed modification is as follows:

**Real time qualitative PCR and computational molecular simulation docking model**

We detected the relative mRNA expression levels of ABL1, CDK1, FYN and AURKA between BCa cell UMUC-3 and normal bladder epithelial cell SV-HUC-1 by PCR. The UMUC-3 cells (CL-0463, Procell, Wuhan, China) were cultured in complete MEM medium (KGM41500S-500, Keygen BIO, Jiangsu, China) and the SV-HUC-1 cells (IM-H068, Immocell, Xiamen, China) were cultured in a dedicated medium (IM-H068-1, Immocell, Xiamen, China). These cells were cultured in an incubator with 5% carbon dioxide, and the temperature was maintained at 37℃. The PCR primers for the four hub genes (ABL1, AURKA, CDK1, and FYN) were designed using Primer 5 software. RNA was extracted from the cells using Trizon reagent (CW0580S, Cwbio, Jiangsu, China), and 0.2 mL chloroform was added for each 1 mL of Trizon. mRNA was extracted using the mRNA ultrapure extraction kit (CW0581M, Cwbio, Jiangsu, China), and miRNA was extracted using the miRNA extraction kit (CW0627S, Cwbio, Jiangsu, China). The OD260/280 ratio of mRNA and miRNA was determined using a UV-visible spectrophotometer. cDNA was synthesized using HiScript II Q RT SuperMix for qPCR (+gDNA wiper) (R223-01, Vazyme, Nanjing, China), and fluorescence quantitative PCR was performed using a fluorescence PCR instrument. The forward and reverse primers were as follows: β-actin (TGGCACCCAGCACAATGAA and CTAAGTCATAGTCCGCCTAGAAGCA); AURKA (CACCCAAAAGAGCAAGCAGC and TTACCCAGAGGGCGACCAA); ABL1 (TGAAGCCGCTCGTTGGA and AGACTGTTGACTGGCGTGATGT); CDK1 (GGGGTCAGCTCGTTACTCAAC and GCCCAAAGCTCTGAAAATCCTG); FYN (TACCAAATCTTGTGGACATGGCA and GATGGGGAACTTTGCACCTTG).

Molecular docking of Res and AURKA proteins was conducted using Autodock software to further analyze their interaction. Firstly, the 3D structure of AURKA was selected from the Protein Data Bank (PDB) database (https://www.rcsb.org/). Due to the excessive flexibility of some loops in the protein which might lead to low electron cloud density and miss coordinate information, the missing amino acids of the AURKA structure were reasonably reconstructed through the AlphaFold3 database (https://alphafold.ebi.ac.uk/) before analyzing its 3D spatial structure. Subsequently, the 3D structure of the Res ligand was obtained from the PubChem website (https://pubchem.ncbi.nlm.nih.gov/). The selected AURKA receptor and Res ligand were imported into PyMol software, and the system automatically performed some preprocessing such as removing water molecules and metal ions to simplify the calculation. Then, the number of poses were selected on the docking page, and the default active pocket region of AURKA was 10. The substrate molecule was docked into the active pocket region through semi-flexible docking, resulting in 10 docking results. The docking model with the lowest energy was selected as the final docking result.

**Cell counting kit 8 (CCK8) and flow cytometry detection**

10 mg of resveratrol powder (HY-16561, MCE) was dissolved in 2.19 ml of dimethyl sulfoxide (DMSO, Solarbio, Beijing, China) to form a 20 mM stock solution, which was then diluted with MEM complete medium to 0, 400, 600, 800 and 1000 μM for treating UMUC-3 cells for 2 hours, followed by treatment with 0, 50, 100, 150 and 200 μM for 24 hours. The AURKA inhibitor MLN8237 solution (HY-10971, MCE) was prepared into 0, 2, 10, 50, 100, and 150 μM with MEM complete medium to treat UMUC-3 cells for 24 hours. Subsequently, the cells to be tested were collected with a volume of 100 μl per well. 10 μl of CCK8 reagent (KGA317, Keygen BIO, Jiangsu, China) was added to each well, and they were incubated in the incubator for 2 hours. The absorbance of each well was detected at 450 nm with an enzyme marker (SuPer Max3100, Shanghai, China). The cell survival rate was detected by the CCK8 method to calculate the concentrations of Res for 30% inhibition (IC30) and for 50% inhibition (IC50). At the same time, the half-maximal inhibitory concentration of MLN8237 was calculated.

According to the different concentrations of Res, the cells were divided into 0 concentration, IC30 concentration, IC50 concentration and high concentration groups. After treating the cells of the four groups for 2 hours and 24 hours, 1x10^6 cells were collected from each group. The cells were washed twice with 1 ml PBS (G4202, Servicebio, Wuhan, China) at 1500 rpm and the supernatant was discarded. Then, 500 μl of apoptosis positive control solution was added to resuspend the cells, which were incubated on ice for 30 minutes. After adding 1 ml PBS for washing, an appropriate amount of 1×binding buffer was added to resuspend the cells and an equal number of untreated cells were mixed with them. Each sample was divided into 3 tubes of 1.5 ml, and 5 μl Annexin V-FITC and 10 μl PI (AP101-100-kit, MULTI SCIENCES, Hangzhou, China) were added to each tube and gently mixed. After incubation at room temperature for 5 minutes, the apoptosis of the cells was detected by flow cytometry (NovoCyte 2060R, Aisen Gene, China). Next, we used the Cell Cycle Staining Kit (CCS102, MULTI SCIENCES, Hangzhou, China) to detect the cell cycle of the above groups of cells. 1x10^6 cells were collected from each group. The cell suspension was washed by centrifugation at 1500 rpm and the supernatant was discarded. Then, 1 ml PBS was added and centrifuged at 1500 rpm to discard the supernatant. Subsequently, 1 ml DNA staining solution and 10 μl permeabilization solution were added, vortexed for 5-10 seconds to mix, and incubated at room temperature in the dark for 30 minutes. The cell cycle was detected by flow cytometry (NovoCyte 2060R, Aisen Gene, China).

**Immunofluorescence staining and detection of STAT3 pathway genes**

UMUC-3 cells were divided into 0-concentration control group, IC50 Res group, and IC50 MLN8237 group. After 24 hours of culture, the culture dishes of each group were fixed with 4% tissue cell fixation solution (P1110, Solarbio, Beijing, China) for 15 minutes and then washed three times with PBS. After blocking with 5% BSA (A8020, Solarbio, Beijing, China) at 37°C for 30 minutes, the cells were incubated overnight at 4°C with diluted rabbit anti-STAT3 (ET1605-45, HUABIOI, 1/200). After being rewarmed at room temperature for 45 minutes and washed three times with PBS, diluted Cy3 Goat Anti-Rabbit IgG (H+L) (AS007, ABclonal, 1/200) was added and incubated at 37°C for 30 minutes, followed by rinsing with PBS. The specimens were stained with DAPI staining solution (KGA215-50, Keygen BIO, Jiangsu, China), rinsed with PBS, and dried before being sealed. The stained cells were observed and images were collected under a fluorescence microscope (CKX53, Olympus, Janpan). The cell nuclei stained with DAPI appear blue under ultraviolet excitation, while positive expression is indicated by the red fluorescence of the corresponding fluorophore (DAPI excitation wavelength 330-380 nm, emission wavelength 420 nm, emits blue light; CY3 excitation wavelength 510-560 nm, emission wavelength 590 nm, emits red light).

Further, according to the different reagents added, UMUC-3 cells were divided into blank control group, IC30 Res group, IC50 Res group and IC50 MLN8237 group. After 24 hours of culture, RNA was extracted from the collected cells using the ultra-pure RNA extraction reagent (CW0581M, Cwbio, Jiangsu, China), and cDNA was synthesized using the HiScript II Q RT SuperMix for qPCR (+gDNA wiper) (R223-01, Vazyme, Nanjing, China). Fluorescence quantitative PCR was performed using a fluorescence PCR instrument. The reaction steps were as follows: pre-denaturation at 95°C for 30 seconds; denaturation at 95°C for 10 seconds; annealing at 58°C for 30 seconds; extension at 72°C for 30 seconds; 40 cycles. The relative expression levels of the three downstream genes of the STAT3 pathway, cyclinD1, VEGF, and BCL-2 mRNA, were calculated according to the 2-△△Ct method. The forward and reverse primers were as follows: β-actin (TGGCACCCAGCACAATGAA and CTAAGTCATAGTCCGCCTAGAAGCA); Bcl-2 (GAGGATTGTGGCCTTCTTTG and GCCGGTTCAGGTACTCAGTC); Cyclin D1 (GGCGGAGGAGAACAAACAGA and GGCGGATTGGAAATGAACTT); VEGF (GAGGAGGGCAGAATCATCAC and TGAGGTTTGATCCGCATAATCT).

Western Blot (WB) was used to detect the expression differences of AURKA, cyclinD1, VEGF, and BCL-2 proteins. Firstly, 200 μL of cell RIPA lysis buffer (C1053, Applygen, Beijing, China) was added to each well, and the cells were disrupted using a cell disruptor. The samples were then centrifuged at 12,000 r/min at 4°C for 10 minutes, and the supernatant was collected and transferred to a new centrifuge tube for protein quantification. The protein concentration was quantified using the BCA protein quantification kit (E-BC-K318-M, Elabscience, Wuhan, China). The protein samples were denatured and stored at -20°C. Sodium dodecyl sulfate-polyacrylamide gel electrophoresis (SDS-PAGE) was performed for 1.5 hours, followed by constant current transfer at 300 mA for 1-2 hours. The PVDF membrane (IPVH00010, Millipore, USA) was blocked with a special defat dried milk (P1622, Applygen, Beijing, China) and incubated overnight at 4°C. The primary antibodies were: Mouse Anti-GAPDH (HC301, TransGen Biotech, 1/2000) for the internal reference, Mouse Anti AURKA (66757-1-Ig, Proteintech, 1/2000), Mouse Anti Bcl-2 (YM3041, ImmunoWay, 1/1000), Rabbit Anti VEGF (AF5131, Affinity, 1/1000), and Rabbit Anti cyclinD1 (ab134175, abcam, 1/20000). The next day, the PVDF membrane was incubated with the secondary antibodies at room temperature for 2 hours: HRP conjugated Goat Anti-Mouse IgG (H+L) (GB23301, Servicebio, 1/2000) for the internal reference, and HRP conjugated Goat Anti-Rabbit IgG (H+L) (GB23303, Servicebio, 1/2000) for the target. The membrane was washed, soaked in the luminescent solution and imaged using a high-sensitivity chemiluminescence imaging system (Tanon-5200, Tanon, Shanghai, China).

**Statistical analysis**

GraphPad Prism 9.5 software was used for statistical analysis and graphing. All experiments were repeated three times, and the quantitative results were expressed as mean ± standard deviation (X ± S). One-way analysis of variance was used for comparison of quantitative values among multiple groups, with a significance level of α = 0.05. P value < 0.05 was considered statistically significant.
