# Supplementary material for: Resveratrol inhibits bladder cancer proliferation by targeting the AURKA/STAT3 axis: From computational analysis to experimental validation
Source: PLoS One. 2026 Jan 30;21(1):e0342162. doi: 10.1371/journal.pone.0342162 (PMC12857983; doi:10.1371/journal.pone.0342162)
Supplement: Attachment — Submitted filename: Response_to__Reviewers_auresp_2.docx [file pone.0342162.s020.docx]

Response to Reviewers

Dear academic editor and reviewers,

We sincerely appreciate you for your recognition and for providing valuable suggestions that have significantly improved the quality of our manuscript [No.: PONE-D-25-45593R1]. We concur with the position that all citations should be strictly relevant to the research. Following these valuable suggestions, the detailed point-by-point response is as below.

**Comment:**

Authors are requested to remove the reviewer-suggested citations from both Version 1 and Version 2 of the manuscript. PLOS ONE does not encourage the inclusion of citations that are not directly relevant to the study. Inclusion of such citations solely at the reviewers’ request may conflict with journal policy and could negatively impact the manuscript’s evaluation, potentially leading to rejection.

The editor assures that removal of these suggested citations from both versions will not influence the editorial decision on the manuscript.

**Response：**

Thank you for your kind comments on our manuscript. We fully agree with your suggestion that irrelevant citations should not be included in the manuscript. Therefore, we have re-reviewed the opinions of the reviewers and the supplementary content of our manuscript. Compared to the version 1, the second version of our manuscript has deleted 2 references and updated 16 references. Based on your reasonable suggestions, we deleted some references from the discussion section and these deleted references were marked as "[26-30]" in the second version. Since the remaining references are related to the research and they were not designated references by the reviewers, we retained some of the updated references which were used to enhance the rigor and quality of our manuscript and did not further reference the 5 designated references on molecular docking by the reviewer. We rechecked the reference list and ensured that the retained references are not withdrawn or specific papers. For your convenience, the eleven updated and retained references of the second version are listed below. If you have further suggestions on our citations, we are honored to receive your further guidance.

“14.	Zucchi A, Claps F, Pastore AL, Perotti A, Biagini A, Sallicandro L, et al. Focus on the Use of Resveratrol in Bladder Cancer. Int J Mol Sci. 2023;24(5). Epub 20230226. doi: 10.3390/ijms24054562. PubMed PMID: 36901993; PubMed Central PMCID: PMCPMC10003096.

23.	Katsimperis S, Tzelves L, Feretzakis G, Bellos T, Tsikopoulos I, Kostakopoulos N, et al. Circulating Tumor DNA in Muscle-Invasive Bladder Cancer: Implications for Prognosis and Treatment Personalization. Cancers (Basel). 2025;17(12). Epub 20250608. doi: 10.3390/cancers17121908. PubMed PMID: 40563559; PubMed Central PMCID: PMCPMC12190218.

24.	Attanasio G, Failla M, Poidomani S, Buzzanca T, Salzano S, Zizzo M, et al. Histological and immunohistochemical approaches to molecular subtyping in muscle-invasive bladder cancer. Front Oncol. 2025;15:1546160. Epub 20250715. doi: 10.3389/fonc.2025.1546160. PubMed PMID: 40735045; PubMed Central PMCID: PMCPMC12303812.

25.	Huang J, Michaud E, Shinde-Jadhav S, Fehric S, Marcq G, Mansure JJ, et al. Effects of combined radiotherapy with immune checkpoint blockade on immunological memory in luminal-like subtype murine bladder cancer model. Cancer Biol Ther. 2024;25(1):2365452. Epub 20240611. doi: 10.1080/15384047.2024.2365452. PubMed PMID: 38860746; PubMed Central PMCID: PMCPMC11174127.

31.	Song B, Wang W, Tang X, Goh RMW, Thuya WL, Ho PCL, et al. Inhibitory Potential of Resveratrol in Cancer Metastasis: From Biology to Therapy. Cancers (Basel). 2023;15(10). Epub 20230514. doi: 10.3390/cancers15102758. PubMed PMID: 37345095; PubMed Central PMCID: PMCPMC10216034.

32.	Jang M, Cai L, Udeani GO, Slowing KV, Thomas CF, Beecher CW, et al. Cancer chemopreventive activity of resveratrol, a natural product derived from grapes. Science. 1997;275(5297):218-20. doi: 10.1126/science.275.5297.218. PubMed PMID: 8985016.

48.	Wan P, Ren Y, Deng H, Li H. CDCA4 promotes bladder cancer progression by JAK/STAT signaling pathway. J Cancer Res Clin Oncol. 2025;151(2):46. Epub 20250124. doi: 10.1007/s00432-025-06109-w. PubMed PMID: 39856473; PubMed Central PMCID: PMCPMC11762220.

49.	Liu M, Guo J, Liu W, Yang Z, Yu F. Dual Targeting of Aurora-A and Bcl-xL Synergistically Reshapes the Immune Microenvironment and Induces Apoptosis in Breast Cancer. Cancer Sci. 2025;116(7):1823-35. Epub 20250330. doi: 10.1111/cas.70072. PubMed PMID: 40159464; PubMed Central PMCID: PMCPMC12210044.

50.	Chen R, Zhou Z, Meng X, Lei Y, Wang Y, Wang Y. Emerging opportunities to treat drug-resistant breast cancer: Discovery of novel small-molecule inhibitors against different targets. Front Pharmacol. 2025;16:1578342. Epub 20250829. doi: 10.3389/fphar.2025.1578342. PubMed PMID: 40949156; PubMed Central PMCID: PMCPMC12425923.

51.	Zhang L, Guo W, Lu N, Tian Y, Yang J, Wang L. Advances in research on unphosphorylated STAT3: A review. Medicine (Baltimore). 2025;104(30): e43476. doi: 10.1097/MD.0000000000043476. PubMed PMID: 40725945; PubMed Central PMCID: PMCPMC12303475.

52.	Wang J, Liu T, Chen P, Yin D, Zhang H, Qiu X, et al. Pharmacokinetic evaluation of two oral Resveratrol formulations in a randomized, open-label, crossover study in healthy fasting subjects. Sci Rep. 2025;15(1):24515. Epub 20250708. doi: 10.1038/s41598-025-08665-0. PubMed PMID: 40628835; PubMed Central PMCID: PMCPMC12238501.”

**Comment:**

Reviewer #1: All other comments have been addressed except the raw images that yo provided have been merged together. You were asked to provide all the individual images, for each individual concentration in triplicates, that too in deperate jpg/png/tiff format with high resolution, uncropped and unedited, machine/instrument generated images. That means for each dose, there will be 3 replicate images to be seperately sent not clubbed together as you have sent the S1, S2 and S3 raw images.

Additionally, please consult and cite the following important references how to write the methods and results part of molecular docking:

a). DOI: 10.1016/j.insi.2025.100143

b). DOI: 10.1155/joch/5133015

c). DOI: 10.1016/j.micpath.2024.106627

d). From silico to benchtop: cosmosiin as a PD-1/PDL-1 immune checkpoint inhibitor revealed through DFT, network pharmacology analysis, and molecular docking integrated experimental verification.

e). Novel C-3 and C-20 derived analogs of betulinic acid as potent cytotoxic agents: design, synthesis, in vitro and in silico studies.

If possible, you should also carry out the MD SIMULATION analysis in order to validate your docking.

**Response：**

Thank you for your reasonable guidance regarding the image presentation. We provide individual original figures as requested and uploaded them separately as supplementary files. We sincerely appreciate your recommendation concerning the value of Molecular Dynamics (MD) simulations to further elucidate protein-ligand dynamics and binding stability. The current study is focused on the preliminary investigation of the inhibitory mechanism of resveratrol in bladder cancer cells. The molecular docking analysis served as a bridge between our prior database screening and subsequent experimental validation, allowing us to demonstrate from multiple perspectives that resveratrol inhibits bladder cancer cell proliferation via the AURKA/STAT3 axis. We fully recognize that MD simulations can provide deeper mechanistic insights beyond static docking. At the same time, we have explained that the subsequent experiments will focus more on the construction of resveratrol stability and in vivo studies in the discussion section of our manuscript. Your valuable suggestions have provided excellent ideas for our future mechanism research. Moreover, we also thank you for providing five valuable references related to molecular docking methodology. In accordance with explicit instruction of the academic editor and journal that inclusion of specific citations solely at the reviewers’ request may conflict with journal policy, we have not included these citations in our manuscript. Nevertheless, we agree that these important references provide a very solid theoretical foundation for our subsequent exploration of the molecular dynamics between natural substances and proteins, as well as for the detection of protein stability and its interaction with ligands. We carefully read and collected these documents for continuous learning. Once again, we would like to thank you for your valuable suggestions that have helped us improve our manuscript and provided important guidance for subsequent research.

Thank you again for your valuable time and the constructive feedback. We wish you a smooth start to the new year and look forward to hearing from you regarding our manuscript.
